# Interments in the City and Liberties of Philadelphia, from the 14th to the 21st of August

**Published:** 1841-08-28

**Authors:** 


### HEALTH OF THE CITY

INTERMENTS *in the City and Liberties of Philadelphia, from the* 14*th to the* 21*st of August.*

| Diseases. | Adults. | Children. | Diseases. | Adults. | Children. |
|---|---|---|---|---|---|
| Apoplexy, | 1 | 0 | *Brought forward,* | 41 | 59 |
| Cancer of the Stomach, | 1 | 0 | Marasmus, | 1 | 10 |
| Casualties, | 1 | 1 | Morbus Cœruleus, | 0 | 1 |
| Colic, | 0 | 1 | Neglect, | 0 | 1 |
| Consumption of the lungs, | 9 | 3 | Old age, | 3 | 0 |
| Convulsions, | 0 | 12 | Pleurisy, | 1 | 0 |
| Diarrhœa, | 1 | 10 | Purpura Hæmorrhagica, | 1 | 0 |
| Dropsy, | 1 | 0 | Sore Mouth, | 0 | 1 |
| Dropsy of the heart, | 2 | 0 | Small pox, | 2 | 2 |
| —— abdominal, | 1 | 0 | Still-born, | 0 | 12 |
| —— of the head, | 0 | 7 | Suicide, | 1 | 0 |
| —— in the breast, | 1 | 0 | Summer Complaint, | 0 | 33 |
| Disease of Brain, | 1 | 0 | Structure of Œsophagus, | 0 | 1 |
| —— Bowels, | 0 | 1 | Tabes Mesenterica, | 0 | 2 |
| —— Spine, | 0 | 1 | Ulcers, | 1 | 0 |
| Dysentery, | 6 | 8 | Unknown, | 1 | 0 |
| Debility | 0 | 1 | | — | — |
| Effects of Laudanum, | 2 | 0 | Total, 174— | 52 | 122 |
| Fracture of skull, | 1 | 0 | | | |
| Fever, Remittent, | 3 | 1 | | | |
| —— Typhus, | 1 | 0 | | | |
| —— Scarlet, | 0 | 2 | | | |
| Hæmorrhage, | 1 | 0 | | | |
| Hysteria, | 1 | 0 | | | |
| Inflammation of the Brain, | 1 | 3 | | | |
| —— Bronchi, | 0 | 2 | | | |
| —— Lungs, | 1 | 2 | | | |
| —— Stomach and Bowels, | 0 | 2 | | | |
| —— Bowels, | 0 | 1 | | | |
| —— Liver, | 2 | 0 | | | |
| —— Peritonæum, | 3 | 0 | | | |
| Jaundice, | 1 | 0 | | | |
| | — | — | | | |
| *Carried forward,* | 41 | 59 | | | |

Of the above, there were under 1 year, 64

| | | | |
|---|---|---|---|
| From | 1 to 2 | | 37 |
| | 2 to 5 | | 14 |
| | 5 to 10 | | 3 |
| | 10 to 15 | | 0 |
| | 15 to 20 | | 4 |
| | 20 to 30 | | 12 |
| | 30 to 40 | | 13 |
| | 40 to 50 | | 7 |
| | 50 to 60 | | 6 |
| | 60 to 70 | | 5 |
| | 70 to 80 | | 7 |
| | 80 to 90 | | 2 |
| Total, | | | 174 |

Of the above there were 11 from the alms-house, 21 people of colour, and 1 from the country, which are included in the total amount.

